# DAILY HEALTH PROBLEMS, SUBJECTIVE AGING, AND THE MODERATING ROLE OF AWARENESS OF AGE-RELATED CHANGES

**DOI:** 10.1093/geroni/igae098.3269

**Published:** 2024-12-31

**Authors:** Maiken Tingvold, Anna Kornadt

**Affiliations:** University of Luxembourg, Esch-sur-Alzette, Grevenmacher, Luxembourg; University of Luxembourg, Esch-sur-Alzette, Grevenmacher, Luxembourg

## Abstract

Indicators of subjective aging, such as a persons’ subjective age or their perception of accelerated aging, are related to health problems, in that experiencing health issues can lead to more negative subjective aging. This has been shown for longitudinal relationships as well as in daily assessments. In the current study, we were interested in moderators of this relationship. We assumed that on days in which participants experienced more age-related gains, the coupling of daily subjective aging and health problems should be weaker, whereas it should be amplified on days in which participants experienced more age-related losses. A sample of N = 70 participants aged 52 – 75 years (Mage = 62.74, SD = 5.53) reported on their daily subjective age (uni- and multidimensional), accelerated aging, health problems and awareness of age-related gains and losses on up to 14 days of daily diary assessments. Age, gender, education, and baseline health were included as covariates. Multilevel models showed that as expected, the perception of age-related losses increased the impact of daily health problems on multidimensional subjective age and accelerated aging. Contrary to expectations, however, more daily gains also increased the impact of daily health problems on subjective age and accelerated aging. Our findings attest to the importance of both the perception of age-related gains and losses in daily life, and show that irrespective of valence, perceiving changes as age-related might be influential for the interpretation of daily experiences and their impact on developmental outcomes.

